# Transition practice for primary immunodeficiency diseases in Southeast Asia: a regional survey

**DOI:** 10.3389/fimmu.2023.1209315

**Published:** 2023-07-13

**Authors:** Chee Mun Chan, Amir Hamzah Abdul Latiff, Lokman Mohd Noh, Intan Hakimah Ismail, Intan Juliana Abd Hamid, Woei Kang Liew, Youjia Zhong, Narissara Suratannon, Rapisa Nantanee, Fatima Johanna Santos-Ocampo, Mary Anne R. Castor, Le Nguyen-Ngoc-Quynh, Anh Thi Van Nguyen, Huyen Thanh Thuc, Nguyen Minh Tuan, Dina Muktiarti, Rizqi Amalia, Sophâl Chean, Lytheang Try, Adli Ali

**Affiliations:** ^1^ Department of Pediatric, Faculty of Medicine, Universiti Kebangsaan Malaysia, Kuala Lumpur, Malaysia; ^2^ Research Center, Hospital Tunku Ampuan Besar Tuanku Aishah Rohani, Universiti Kebangsaan Malaysia (UKM) Specialist Children’s Hospital, Kuala Lumpur, Malaysia; ^3^ Allergy and Immunology Centre, Pantai Hospital, Kuala Lumpur, Malaysia; ^4^ Malaysian Patient Organization for Primary Immunodeficiencies (MYPOPI), Shah Alam, Selangor, Malaysia; ^5^ Clinical Immunology Unit, Department of Paediatrics, Faculty of Medicine and Health Sciences, Universiti Putra Malaysia, Serdang, Malaysia; ^6^ Primary Immunodeficiency Diseases Group, Department of Clinical Medicine, Institut Perubatan & Pergigian Termaju, Universiti Sains Malaysia, Kepala Batas, Pulau Pinang, Malaysia; ^7^ Rheumatology and Immunology Service, Department of Pediatric Medicine, KK Women’s and Children’s Hospital, Singapore, Singapore; ^8^ Department of Pediatrics, Yong Loo Lin School of Medicine, Kent Ridge, National University of Singapore, Kent Ridge Country, Singapore; ^9^ Center of Excellence for Allergy and Clinical Immunology, Division of Allergy, Immunology and Rheumatology, Department of Pediatrics, Faculty of Medicine, Chulalongkorn University, Bangkok, Thailand; ^10^ King Chulalongkorn Memorial Hospital, The Thai Red Cross Society, Bangkok, Thailand; ^11^ Section of Allergy/ Immunology, Department of Pediatrics, Makati Medical Center, Makati, Philippines; ^12^ Division of Allergy and Immunology, Department of Pediatrics, College of Medicine - Philippine General Hospital, University of the Philippines Manila, Manila, Philippines; ^13^ Stem Cells Transplantation Centre, National Children’s Hospital, Hanoi, Vietnam; ^14^ Allergy - Immunology - Rheumatology Department , National Children’s Hospital, Hanoi, Vietnam; ^15^ Allergy and Clinical Immunology Center, Vinmec International Hospital, Hanoi, Vietnam; ^16^ Department of Paediatrics, Children’s Hospital 1, Ho Chi Minh, Vietnam; ^17^ Department of Child Health, Faculty of Medicine, Universitas Indonesia - Cipto Mangunkusumo Hospital, Jakarta, Indonesia; ^18^ Department of Pediatric Hemato-Immunology, National Pediatric Hospital, Phnom Penh, Cambodia; ^19^ Institute of IR4.0, Universiti Kebangsaan Malaysia, Bangi, Malaysia; ^20^ Infection and Immunology Health and Advanced Medicine Cluster, Universiti Kebangsaan Malaysia, Kuala Lumpur, Malaysia

**Keywords:** primary immunodeficiencies, transition practice, challenges, opinion, Southeast Asia (SEA)

## Abstract

**Introduction:**

With increased diagnostic capabilities and treatment modalities in the field of primary immunodeficiencies (PID), many pediatric patients survive beyond childhood and experience a change of care to the adult-oriented healthcare system. Unfortunately, the transition pathways for PID are less clearly defined, resulting in deterioration of quality of care in adulthood. Hence, this is the first regional study to address PID clinicians’ opinions on practices and challenges of transition care in 7 Southeast Asia (SEA) countries.

**Methods:**

We adopted a cross-sectional study design through an online survey platform to enquire opinions of transition practices from expert representatives in 7 SEA countries.

**Results:**

Regionally, 3 out 7 countries reported having no practice of transition care. Among cited challenges were reluctant adaptation by patients and caregivers to unfamiliarized adult healthcare systems, inadequate ratio of adult immunologists to patients and lack of facilities for transfer.

**Discussion and conclusion:**

Our study provides evidence to advocate policy makers on the importance of standardized integration of transition practice towards betterment of transiting PID patients into adulthood.

## 1 Introduction

Inborn error of immunity (IEI) describes a heterogeneous group of over 400 rare inherited disorders characterized by increased, poor or absent immune system function (1). It is estimated that the overall prevalence of these disorders is approximately 1 in 1000 to 1 in 5000 (2). This umbrella term encompasses many of the rare disorders, including primary immunodeficiency diseases (PID), which often present with infection susceptibility during pediatric age, and may later be associated with autoimmune, inflammatory and malignant complications (3–5). With the advancement of science and medicine, the diagnostic capabilities and available treatment modalities have improved over the years, leading to much earlier detection of PID. One successful diagnostic modality is the T-cell receptor excision circle assay, which has the potential for earlier detection of severe combined immune deficiency (SCID) at birth from Guthrie dried blood spot samples (6). This remarkable milestone allows for earlier curative intervention, resulting in better outcomes and survival among neonates. If left undiagnosed, SCID is associated with almost 100% mortality within the first 2 years of life (6). As a result of SCID newborn screening and other advances, increasing numbers of patients are surviving into adulthood every year (2). According to a report by the European Society for Immunodeficiencies (ESID) Registry in 2013, 5.45% of all registered PID patients were aged 65 and above, and this figure was projected to increase in the coming years (3). Consequently, there has been greater emphasis by medical professionals on how to provide adequate support and management for PID patients, particularly during their transition to adult healthcare services.

Adolescence is a transitional period between childhood and adulthood that typically occurs between the ages of 10 and 20 years (or up to 24 years for young adults). Importantly, recent evidence suggested that 10% of adolescents are affected by chronic health conditions that require ongoing care in adulthood (7). This phase is commonly referred to as the transition of care, defined as a purposeful, planned process that addresses the medical, psychosocial, and education/vocational needs of adolescents and young adults with chronic physical and medical conditions as they move from child-centered to adult-oriented healthcare systems (3). To date, there are well-established guidelines for other immune-mediated conditions that arise in childhood and continue into adulthood, such as rheumatic diseases, asthma and allergies (2). Unfortunately, despite the increasing awareness and registries of the disease, the transition pathways for PID have been less clearly defined and emphasized (3, 4). Given the maturation process of young adults and the social and personal changes that grant them autonomy to decide on their health, it is important to establish a patient pathway of transition of care. In 2017, the International Patient Organization for Primary Immunodeficiencies (IPOPI) highlighted the importance of developing such a pathway to guide young adults to adult services, improve patients’ compliance with treatment, and reduce unnecessary costs due to health damage (3).

There are several reasons cited for the delayed availability of transition guidelines, including challenges faced by patients, caregivers, and physicians. The complexity of this relationship stems from the strong connections, trust, and familiarity that are nurtured within the triad of care. The European Reference Networks on Rare Immunodeficiency, Autoinflammatory and Autoimmune Disease (ERN-RITA) survey reported that the problems commonly arise for PID adolescents during the transfer of care to an adult team, rather than due to the nature of PID itself in adulthood (2). As adolescents gain autonomy in making decisions regarding their health, it is imperative to assess their understanding of the disease, medication, compliance with treatment, and their preference for transition centers. This is especially important when they begin working, as it ensures that their expectations for adult services are met, and their concerns are well addressed.

Regrettably, the duration of the transition process to adult care remains a topic of disagreement among healthcare professionals, with no widely accepted consensus on the optimal time frame (6). The lack of nationally recognized, disease-specific transition guidelines serves as a significant barrier to the efficient transition of care, hindering the quality and continuity of care for patients. Secondly, according to the PID Life Index, data revealed that in certain countries, the field of immunology is still considered novel, leading to a paucity of immunologists and a limited number of specialized adult care centers, which in turn hinders the efforts to provide optimal transition care (8). This deficiency in specialized areas has resulted in only five out of 55 countries offering a comprehensive national network for adult care coordination. In the Asian region, data from the PID Life Index indicated that out of thirteen countries surveyed, nine countries reported a lack of access to transition care (8). This finding raises concerns regarding the provision of care for adult patients with PID, who may experience a lower quality of life (QOL) and reduced life capacity when compared to pediatric patients. Hence, successful transition from pediatric to adult services is a crucial element of clinical care for chronic conditions, as it has been shown to predict treatment adherence and medical outcomes.

Currently, there is a paucity of literature on the optimal practices for transitioning pediatric primary immunodeficiency patients to adult care. A survey conducted by Zoya and colleagues revealed that 59% of PID providers in the United States expressed dissatisfaction with their current transition practices, while 46% identified inadequate training as a primary obstacle in addressing transition issues (9). As a result, recent efforts have recognized the call by the IPOPI to incorporate transition care at an earlier stage, in light of the early detection of PID and the longer life expectancy of these patients (3). Recent advancement in diagnostic tools have enabled Southeast Asian (SEA) countries to detect PID cases with greater accuracy, ranging from 0% to 35%, albeit still lower compared to developed countries (10, 11). In the last decade, emerging efforts to transition care were seen in several countries like Indonesia, Thailand, Singapore, and Vietnam, but little was known about the challenges and knowledge gaps existed from this new practice. Thus, our study represents the first attempt to gather expert opinions on the practices and challenges of transition care in these SEA countries, including Malaysia, Cambodia, and the Philippines. We aim to address this gap and provide evidence to policymakers on the importance of developing a comprehensive regional/national framework for transition care. This framework will enable a smoother transition of young patients with PID into adulthood, thus improving their long-term health outcomes.

## 2 Materials and methods

### 2.1 Study design

This study employed a cross-sectional design to survey pediatric clinicians providing care for PID patients in Southeast Asia (SEA) between January and April of 2023. Our study aimed to identify at least two experts from each participating country to serve as the country’s representatives. To achieve this, we disseminated our invitation through professional and organizational networks *via* email. The inclusion criterion was pediatric clinicians who are currently practicing and actively managing PID cases in their respective countries. We distributed an online survey *via* email to all experts who had agreed to contribute their opinions on the transition practices of PID in their respective countries.

### 2.2 Data sources and survey structuring

The survey was developed based on literature reviews, followed by focus group discussions involving pediatric immunologists and patients’ representatives from Malaysia. A pilot validation of the survey was then conducted with two Malaysian experts to test its face and content validity, resulting in the final version used in this study.

The survey employed a stratified structure, which focused on three domains, namely (i) patients, (ii) caregivers, and (iii) healthcare providers. Experts were requested to provide their professional opinion on the challenges faced by these three parties concerning transition care. The survey questions were used as a foundational framework to guide experts toward the study’s intended direction. In order to accommodate cultural and socio-economic variations across the seven SEA countries, experts were provided with the option to skip any questions deemed irrelevant. Furthermore, experts were given the opportunity to provide additional remarks to further enhance our understanding of the current state of PID care in their respective countries, which will be thoroughly discussed in the upcoming section. To maintain anonymity among the panel of experts and prevent bias in their opinions, all experts were required to complete the questionnaire themselves and return it directly to the researcher.

### 2.3 Data analysis

The study utilized descriptive analysis to examine the data obtained from the survey. The results were presented as n (%) where n represented the total number of experts who responded to each domain. The frequency of each domain was evaluated individually and presented in tables and figures for comparative purposes. Generally, the analysis focused on the overall opinions of experts across SEA countries rather than individualized comparisons based on each country’s practice.

### 2.4 Ethical considerations

This study was approved and supported by the Secretariat of Research and Innovation Universiti Kebangsaan Malaysia (UKM) (Project code: UKM PPI/111/8/JEP-2023-120). The study was conducted in full compliance with ethical principles outlined in the Declaration of Helsinki and Malaysian Good Clinical Practice Guideline.

## 3 Results

### 3.1 Experts’ demographics

Our study engaged a total of 18 pediatric PID experts, who were affiliated with 14 healthcare centers spanning across seven countries in the SEA region. Among the recruited experts, half of them (50%) reported to have treated fewer than 50 patients in their service. According to our analysis, a majority of experts (61.1%) from Indonesia, Thailand, Vietnam, Singapore, and Malaysia reported that they transferred their pediatric PID patients to adult immunologists. However, the number of patients transferred was low, with less than 5 patients per year. On the contrary, the majority of experts from Malaysia and the Philippines reported that they remained as primary care providers to PID patients and only referred them to adult specialists depending on underlying issues. In Cambodia, the management of PID patients was unique as they were handled by pediatric hematologists. As a result, they were transferred to adult hematologists when they reached adulthood. Generally, the majority of experts (61%) transferred or referred their patients to adult care between 18 to 20 years old. The demographic information is summarized in **Table 1**.

**Table 1:** Demographics of expert representatives from Seven Southeast Asia (SEA) countries.

Practice of transition care ^a^	Number of experts, n (%) N=18
Yes	11 (61.1)
No	7 (38.9)
Number of PID patients treated in service	
<50	9 (50.0)
51 to 100	4 (22.2)
101 to 200	2 (11.1)
>200	3 (16.7)
Number of patients transferred to adult immunologist per year	
<5	11 (61.1)
5 to 10	0
11 to 20	0
>20	0
N/A	7 (39.9) ^b^
Age to start transition/referral process (years)	
9 to 11	0
12 to 14	1 (5.6)
15 to 17	1 (5.6)
18 to 20	12 (66.6)
N/A	4 (22.2) ^b^
Referral options in adult care	
Adult immunologist	11 (61.1)
Adult subspecialized internal medicine specialist	3 (16.7)
Adult hematologist	2 (11.1)
N/A	2 (11.1) ^b^

a Indicates practice of transitioning patients to dedicated adult immunologists and adult healthcare center.

b Indicates that transition care to adult immunologists is not practiced by the expert.

Descriptive analysis was used to compare the demographic information in 7 SEA countries. The results were expressed in n (%), where n represented the number of experts.

### 3.2 Transition preparation and process

**Figure 1:**
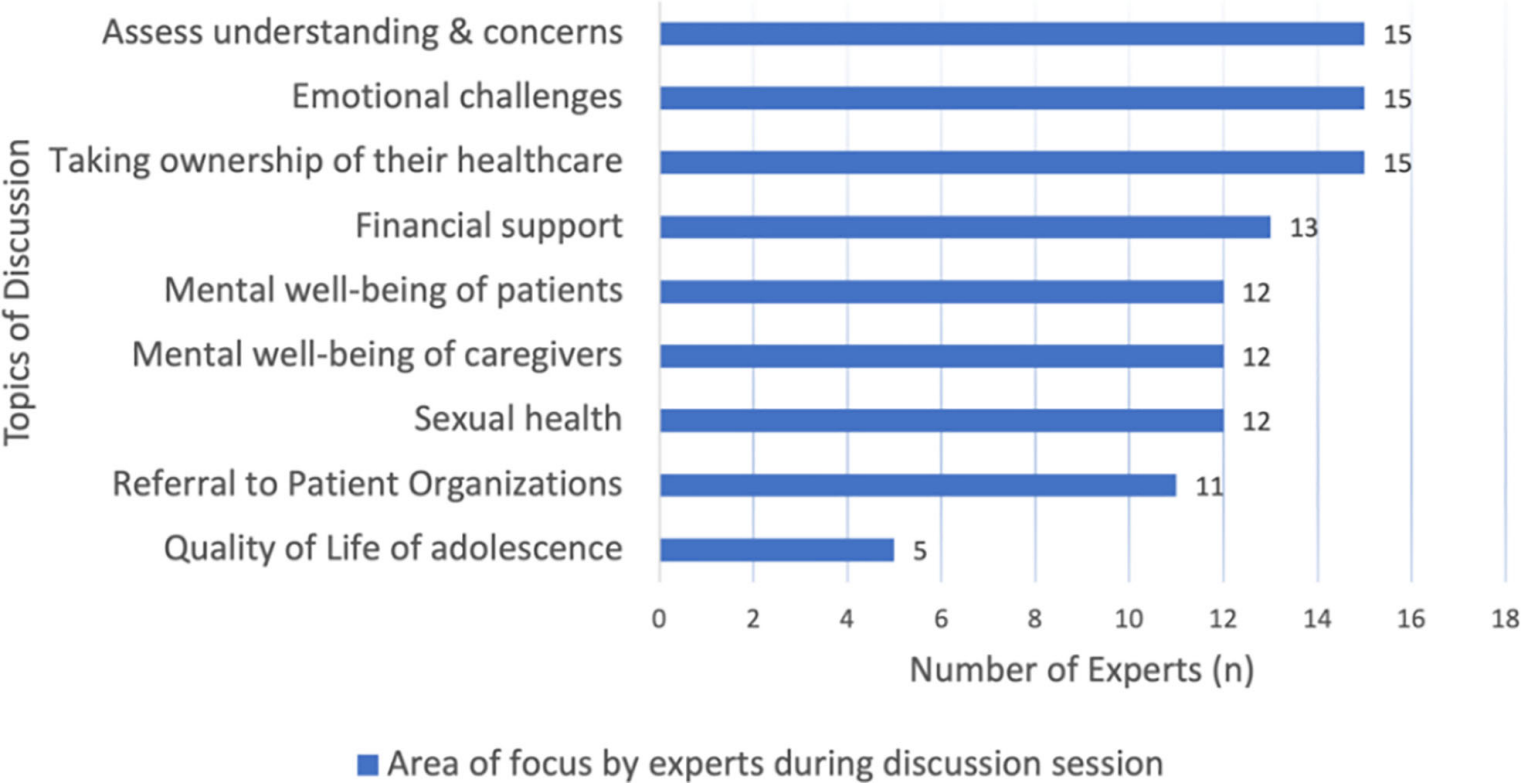
Discussion topics with patients and caregivers during transition and referral process in Southeast Asia (SEA). We used descriptive analysis to compare the frequency of discussion topics by PID experts in SEA. The results were expressed in n (%), where n represented the number of experts.

**Figure 2:**
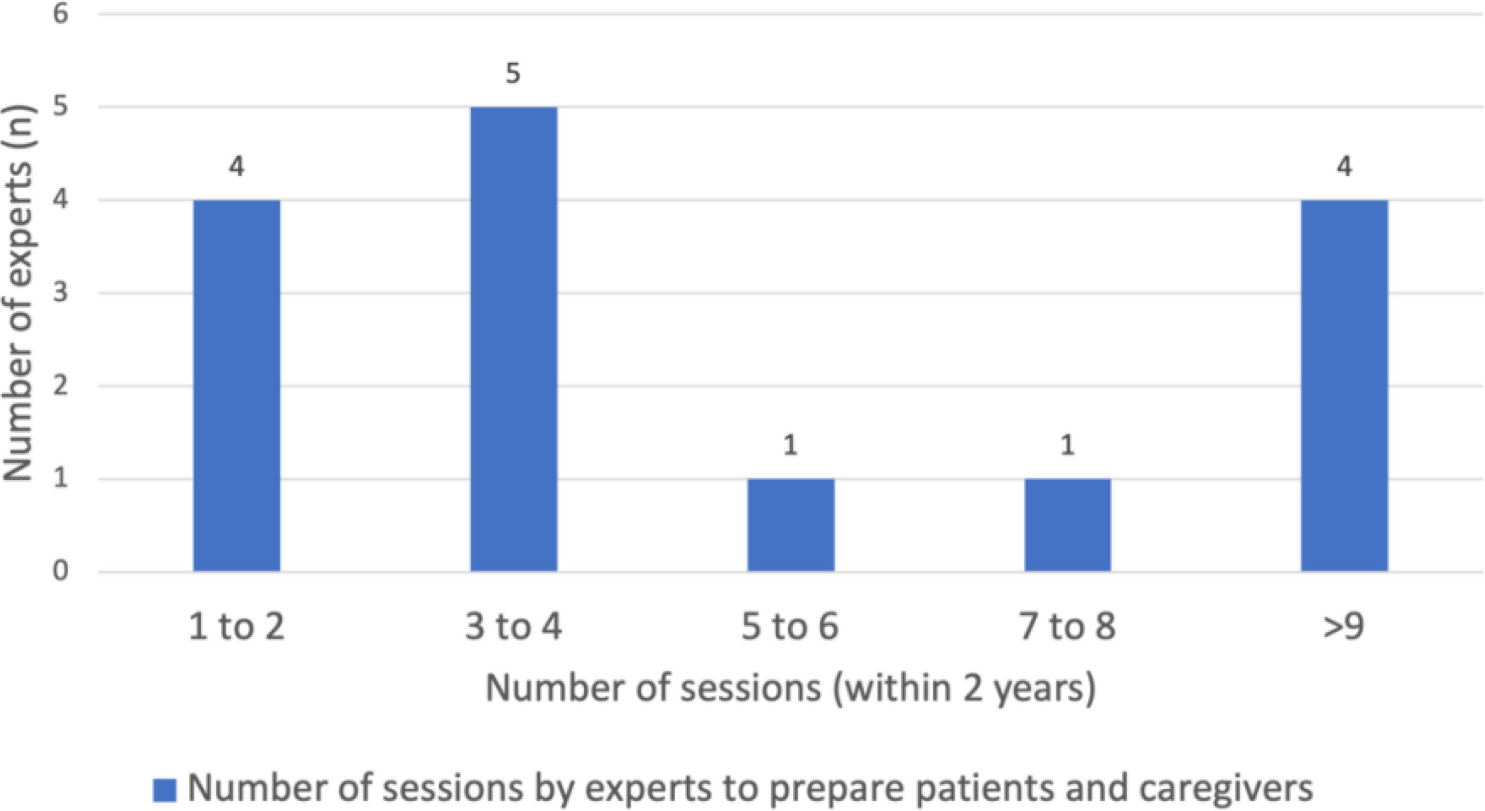
Number of sessions required by experts to gauge patients’ understanding and readiness during transition process in Southeast Asia (SEA). We used descriptive analysis to compare the number of sessions practiced by SEA experts. The results were expressed in n (%), where n represented the number of experts.

Prior to actual transfer or referral of PID patients to adult care, the experts engaged in discussions with PID patients and caregivers on various health-related and psychosocial topics, depicted by **Figure 1**. Firstly, it is commendable that the majority of experts (83%) assessed the understanding and concerns of their patients and caregivers on transition care. Experts found that multiple sessions were required to gradually impart information and gauge patients’ understanding of the transfer of care process. This was seen as an essential step in ensuring good compliance to treatment and maintaining the quality of care provided in adult services. Hence, the responses of the 15 experts were further stratified, revealing that a majority of them (33.3%) conducted 3 to 4 sessions over a period of 1 to 2 years to impart information and assess patients’ understanding (**Figure 2**). In addition, it is noteworthy to mention that over 80% of the experts held the belief that PID adolescents should strive for independence from their caregivers and assume responsibility for their own health at an early stage. Approximately 50% of the experts initiated the education process with their patients at the age of 12 to 14 years, emphasizing the importance of recognizing warning signs for timely and effective treatment. Thirdly, a significant proportion (83%) of the experts also acknowledged and addressed the emotional challenges faced by their patients during the transitional process. While 56.3% of the experts acted as the primary counselors to assess the mental health of their patients during the transition process, some experts referred PID adolescents to their psychiatrist colleagues if emotional/mental red flags symptoms such as depression, panic attack and anxiety were observed. Regrettably, the quality of life of these PID adolescents during the transition phase was poorly addressed, as only 27.8% of the experts specifically looked into this matter.

Caregivers also played an integral role in ensuring that transition to adult services were well accepted by their children. It is worth noting that more than half of the experts showed concern for the mental well-being of caregivers. It is irrefutable that parents of children with PID may develop emotional and psychosocial stress due to various challenges, such as financial barriers related to the sustainability of treatment costs as the children age (38.9%), logistical and support issues related to transitioning to an adult hospital (27.8%), frequent absenteeism from work (16.7%), and reduced insurance coverage as the child reaches adulthood (22.2%). As a result, a significant proportion of the experts (ranging from 61% to 72%) advocated for referral or registration of the patients and their caregivers with relevant patient organizations to access mutual support and financial assistance. This was deemed necessary to alleviate the financial and emotional burden on the caregivers and facilitate a seamless transition process.

### 3.3 Challenges of transition care in Southeast Asia (SEA)

**Figure 3:**
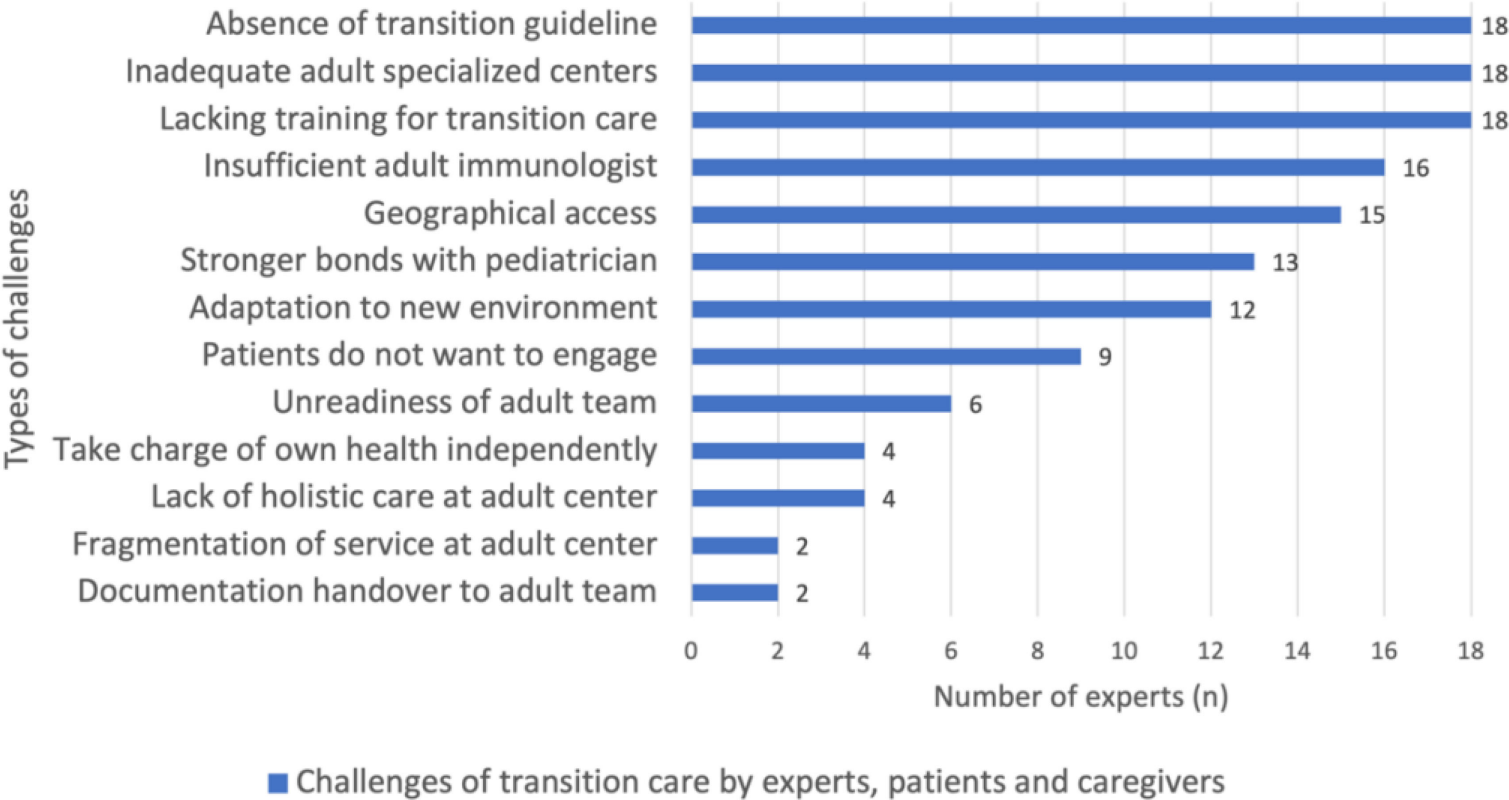
Transition challenges by patients, caregivers, and experts in SEA. We used descriptive analysis to compare the frequency of challenges faced by PID patients, caregivers, and experts in SEA. The results were expressed in n (%), where n represented the number of experts.

All SEA PID experts reported the lack of transition guidelines for transfer of care to adult services as one of the main challenges, seen in **Figure 3**. On top of that, 100% of the surveyed experts in SEA identified inadequate adult specialized centers and training for transition of care as additional challenges in their practice of transition care. Next, about 90% of experts cited insufficient adult immunologists in their countries. Of note, it was found that Cambodia reported not having immunologists who specifically managed PID cases in the country. Among the remaining countries, Malaysia, Singapore, and Vietnam reported having less than 10 adult immunologists available for referral. Thailand and Indonesia reported a slightly better situation, with 20 and 50 adult immunologists available, respectively. Uniquely in the Philippines, immunologists were trained in a joint pediatric and adult immunology fellowship, thus allowing them to provide comprehensive care to PID patients from pediatric to adulthood. As a result, the Philippines experts did not see the lack of immunologists as a limiting factor since immunologists were capable of taking care transitioning pediatric patients to adulthood, despite not having adult internal medicine background. Vice versa, in cases where there were no immunologists with pediatric background, specialized adult medicine immunologists will manage PID pediatric patients with referral to other pediatric colleagues for the rest of the medical care until adulthood.

Approximately 50% of experts opined that the reluctance of patients to engage with adult teams due to the strong bond fostered with their managing pediatricians hindered the transition process. Additionally, some experts noted that adolescents were not always receptive to changes in their care, and the transition to the new environment in adult hospitals and wards could also be daunting for them.

The majority of experts (80%) concurred that caregivers encountered geographic barriers in accessing adult healthcare facilities, which were typically concentrated in urban areas. Experts also expressed concerns that some caregivers were hesitant to transfer their children to adult services due to accessibility issues, which were not financially sustainable in the long run. Moreover, a minority of the experts (22.2%) reported that caregivers expressed their dissatisfaction with adult care, citing the lack of comprehensive and holistic care provided in adult centers.

## 4 Discussion

Despite the increasing global focus on the transition of adolescents with childhood-onset chronic diseases from pediatric to adult healthcare systems, many countries in Asia remain inadequately equipped to manage this process. According to a study conducted in Japan, it was found that around 95.7% of patients with certain chronic diseases that originated during childhood, with the exception of malignancies, managed to survive till adulthood (11). However, these adult patients often face complications possibly attributed to various factors such as age-related changes in therapeutic areas, poor treatment adherence and the development of other non-communicable diseases. In response, the Japan Pediatric Society published a consensus statement in 2014 titled “Proposal for Transitional Care for Patients with Childhood-Onset Diseases’’ based on the six core elements recommended by The American Academy of Pediatrics (AAP), The American Academy of Family Physicians (AAFP) and the American Board of Internal Medicine (12). The six core elements included: (i) establishing the transition policy; (ii) tracking and monitoring transition progress; (iii) assessing the patient’s readiness for transition; (iv) developing the transition plan with a medical summary; (v) transferring the patient; and (vi) completing the transfer and following up with patient and family (13). However, to date, after a decade since the establishment of the consensus, Sakurai and colleagues reported that transition support was still limited due to various barriers involving patients, caregivers, and healthcare providers whereby only a few departments and individuals were dedicated to the transition process (12). To ensure synergy among the triad of relationships involving patients, caregivers, and healthcare providers, it is imperative to adopt a shared patient-centered and family-centered decision-making process among pediatric and adult teams to help make the transition process less stressful for patients and caregivers (4). Thus, our study emphasized the pressing need to comprehend the current state of transitional care and obstacles to the transition of pediatric patients with PID in Southeast Asia.

Firstly, our findings coincide with previous research that has shown the transition process for PID pediatric patients in Southeast Asia often occurs during late adolescence, typically between the ages of 18 to 20 years. However, the process itself, from preparation to actual transfer, often occurs within a short time frame of one to two years, leaving little adjustment time for patients and caregivers to become familiar with adult services (2). This compressed timeline may stem from clinicians’ preconceived notions about the appropriate age for transition, rather than considering patients’ readiness for the transition process (14). It is imperative to assess patients’ readiness to determine the optimal timing for transition, as this can predict health-related outcomes and development of self-management skills toward their chronic illness. Notably, a European study highlighted that patient unwillingness to engage with the transition process represents a significant weakness of the program, a finding with which our study agrees (2). The APP, AAFP and American College of Physicians (ACP) recommend initiating the transition process by age 12, providing ample time to empower adolescents with self-management skills and anticipating eventual transfer of care by age 18 (15). The Italian consensus supports this notion, stating that the transition process should begin at least by age 14 to allow sufficient time to educate adolescents on coping mechanisms related to the long-term psychological effects of suffering of chronic diseases such as PID (4). Malaysia’s experts generally concurred with the findings of Fegran and colleagues that patients and caregivers generally found the sudden transition process to be the most unsatisfactory (16). In the Malaysian healthcare system, pediatric patients who reach the age of 18 and above are required to transfer to adult services for continuation of care, including intravenous immunoglobulin (IVIG) infusion, despite remaining under the primary care provider who manages their condition (17). However, our study found that the policy is implemented immediately upon them reaching 18 years old, leaving little time for preparation and adjustment. Consequently, PID adolescent patients may feel unprepared and unwanted as they move from the familiar pediatric ward to an unfamiliar adult ward, which may result in dissatisfaction with the transition process.

Remarkably, the Philippine experts did not view transitioning of care of PID patients as a great challenge because immunologists take care of PID patients from childhood to adulthood. The need for transition of care is with regards to their co-managing subspecialties, such as pulmonology, infectious disease, rheumatology, neurology. Thus, the Philippines has distinguished itself from other Southeast Asian countries by being the only country establishing dedicated adolescent medicine clinic services aimed at supporting adolescents in managing chronic illness and achieving the transition process objectives (18). Briefly, in the Philippines, the recognition of adolescent medicine as a distinct service date back to the 1980s. However, it was not until 2015 that significant attention was devoted to the health of this demographic. This shift in focus was largely attributable to the efforts of medical organizations, such as the Society of Adolescent Medicine of Philippines (SAMPI) which spearheaded a series of educational summer camps aimed at promoting awareness of the unique health concerns affecting adolescents. In addition, the tertiary level government hospital also recognizes the importance of adolescent medicine by providing opportunities for trainees to pursue adolescent medicine fellowships at the Philippines General Hospital with the hope to expand the reach of adolescent services to provincial hospitals (18). Therefore, it is highly likely that the readiness of patients has a significant impact on the quality of care they receive and their health prognosis in adulthood. In light of practices in Western countries, it may be beneficial to adopt transition readiness scales such as the Transition Readiness Assessment Questionnaire (TRAQ) as a tool to assess the developmental process of healthcare transition readiness among adolescents in SEA (15).

It has been observed that adjusting to a new environment can be a challenging experience, especially when patients have to let go of familiarity without knowing what the future holds (16). In the context of the transition process in SEA, we found that one of the challenges faced by patients was their reluctance to engage with the adult team due to the strong bonds they had developed with the pediatric team. With diagnosis of PID commonly detected in early childhood, this means that the relationship between patients and the pediatric clinician starts early in the patients’ life and gradually evolves into a trusted professional relationship (16). The ambience in pediatric wards tends to be friendlier and more welcoming, creating a more personalized and “homely” experience for patients (16). On the contrary, the doctor-patient relationships in adult hospital care were different as the adult team was perceived as impersonal and disease-focused, creating barriers to establishing a good rapport and relationships with patients. Hence, we concur with a study in Hong Kong, which reported that “Do not want to change” was the only significant barrier preventing adolescents and parents from considering transition care (19). In addition, the lack of comprehensive care also impeded patients from willingly transitioning to the adult team (2). The healthcare personnel in adult wards were often described as “busy and superficial”, which left them ill-equipped to handle adolescent patients and inadvertently created a sense of being a hindrance among adolescents. As a result, adolescents with PID may encounter a decline in the quality of healthcare as they transition to adulthood. This situation is reminiscent of a report in the rheumatology domain where 52% of patients experienced an unsuccessful transition from pediatric to adult care in Canada (20). Therefore, to facilitate the transition process between patients and the adult team, it is encouraged to have multiple discussions or joint clinics to build good rapport and relationship (2). Similar to many studies, all of our SEA experts agreed that accompaniment, defined as a period when the adolescent is followed-up simultaneously by pediatricians and adult specialists is essential in this process (4). This is because meeting with both the physician and non-physician members of the adult team, including psychologists, social workers, peer advocates, and nurse educators, prior to transfer, can help bridge the clinical care gap between pediatric and adult care; promoting patient familiarity that can ultimately lead to a successful transfer when the patient is ready (21). Having multiple discussions with the adult team can also provide PID patients with extensive transition information, thus preparing them for the transition of care. In a study conducted by Lilian and colleagues, it was found that 91.9% of adolescents did not receive any transition information from their doctors, and this was postulated as a major obstacle for patients to accept transition care (19). Insufficient information during the transition process can be a daunting experience for adolescents, particularly when entering an unfamiliar adult service, especially since PID has been reported to have a strong negative effect on both the physical and mental domains of quality of life (QOL) (22). Regrettably, there is a dearth of research in SEA that focuses on assessing the QOL of PID patients during adolescence, and only a small number of experts in the region have investigated this area. A meta-analysis by Peshko and colleagues reported that QOL in children and adults with PID is more adversely impacted compared to healthy individuals and patients with other chronic conditions such as diabetes mellitus and juvenile idiopathic arthritis (23). Despite the known effect, none of the experts used established tools, such as the common variable immunodeficiency (CVID)_QOL questionnaire, to evaluate the QOL of adolescents with PID (24). Thus, we agree to the emphasized notion on the importance of QOL assessment in PID patients by using developmentally appropriate and validated tools according to age groups to promote better understanding of disease processes and health outcomes (23).

Caregivers of PID patients also have a crucial role in facilitating a smooth transition process. Often, in some cases, caregivers of adolescents with chronic diseases tend to become overly involved in deciding an adolescent’s ability to be autonomous in their own health (16). Research has shown that caregivers may be hesitant to allow their children to be autonomous in their own health due to concerns such as financial issues, treatment adherence, and logistical issues with follow-up appointments (15, 25). Therefore, healthcare providers should reassure caregivers by reinforcing supportive roles in preparing their children for independence. In line with regional studies in SEA, we found that financial difficulty remained as one of the challenges faced by caregivers in caring for PID patients (26). For example, financial hardship is a prevailing issue among the population of Vietnam, with a considerable number of rural households in the Northern region experiencing debt due to healthcare costs. Specifically, approximately 60% of these households are affected, which can lead to a destitution of care. In Vietnam, after the government’s efforts of amending the Law on Health Insurance in 2015, the coverage rate of health insurance has increased from 70% to 92%, with payment varying depending on age and degree of economic hardship and disability (25, 27). Patients from poor families or disabled patients or children under 6 years old were entitled to 100% insurance while the remaining subjects were refunded up to 80%. As a result, our Vietnamese experts reported that national health insurance has eased the financial burden for many pediatric PIDs in terms of treatment and hospitalization costs. As medical costs increase with age, patients with lower socioeconomic status may struggle to afford necessary treatments, including those requiring higher dosages based on body weight. This disparity in access to care can result in poorer health outcomes for low-income patients compared to their wealthier counterparts. Furthermore, despite the universal health coverage, many insured patients have reported that the insurance payment provided by the government is insufficient to cover other expenses such as logistic expenses, including travel and accommodation costs for caregivers when accompanying patients to adult hospitals (16). Therefore, the practice of rural patients traveling to urban areas to seek better healthcare services in the adult department increases the risk of patients facing destitution of care. In Japan, a similar issue was encountered where subsidies for medical expenses for pediatric diseases ceased to be available when the child reached 20 years of age (12). Studies cited that this was a major drawback in patients’ lives as they commonly faced difficulties in achieving financial independence due to low income and low employment rate, possibly attributed to the absenteeism from work for regular treatment and follow up. As a result, patients are not eligible to obtain employer-based health insurance and indirectly increase the burden of caregivers to support their children at the age of retirement (12). This phenomenon is also true in Singapore, whereby experts explained that patients who started working will eventually lose their insurance coverage. One of the solutions to this problem is the role of national patient organizations (16). It is applaudable that majority of SEA experts referred caregivers to PID patient organizations such as the Malaysia Patient Organization for Primary Immunodeficiencies (MyPOPI), Indonesia Primary Immunodeficiencies Patient Societies (IPIPS), Vietnam Primary Immunodeficiencies Patient Support Group (VietPIPS), Philippine Patient Organization for Primary Immunodeficiencies (PhilPOPI), Thai Patient Organizations for Primary Immunodeficiencies (ThaiPOPI) and Club Rainbow Singapore, for mutual support and financial aid (28–33). We posit that peer support can play a crucial role in assisting individuals with chronic illnesses to manage their lives as they transition into adulthood. Besides, the support group can provide a platform for caregivers to share their emotions, experiences and offer psychological support to one another, while also receiving financial aid from the organization (16).

It is widely acknowledged by the experts in SEA that the development of PID transition care guidelines is an imperative measure to ensure the provision of optimal healthcare services. According to a European survey, the lack of national disease-specific transition guidelines was identified by 86% of clinicians as one of the barriers in the effective transition of adolescents to adult care (2). A joint consensus statement by AAP, AAFP and ACP have set goals twice stating “all physicians who provide primary or subspecialty care to young people with special health care need to: (i) understand the rationale for transition from child-oriented to adult-oriented healthcare; (ii) have the knowledge and skills to facilitate the process, and (iii) know, the “if”, “how”, and “when” transfer of care is indicated (34). In the Philippines, where immunologists with pediatric or internal medicine background were trained to manage all PID cases regardless of age, transfer of care is mainly focused on the other subspecialty areas involved in the patient’s care. However, Cambodia experts noted that many adult specialists across different subspecialties, including gastroenterology, hematology, and endocrinology, receive insufficient exposure to PID patients during their training (4). Furthermore, immunology is a relatively new field, and there are no specialized immunologists trained to care for patients with PID (4). This is seen in Malaysia where immunology is not recognized as a subspecialty, resulting in insufficient pediatric and adult immunologists (35). On top of that, most of the adult immunologists are primarily managing allergy and autoimmunity cases (4). Furthermore, the lack of adult specialized centers also presented challenges for pediatric teams and caregivers to facilitate the transition of care. Due to the fact that adult centers are often centralized in urban areas and difficult to access, many pediatricians encounter challenges of transferring care due to the logistical and support issues faced by both the caregivers and patients (2). The majority of SEA experts have concurred that overcoming geographical barriers is a significant factor to be considered in the pursuit of delivering high-quality adult services.

The establishment of transition care guidelines in many pediatric-onset chronic childhood diseases has been greatly acknowledged over the past decades. For instance, The European Alliance of Associations for Rheumatology (EULAR) has outlined transition care guidelines for juvenile-onset rheumatic diseases (36). Despite ongoing efforts to ameliorate the discrepancies in the present provision of transition services, research has identified challenges and formulated standardized recommendations to serve as guidance for rheumatologists when transitioning adolescent patients to adult care. Firstly, recommendation for transitioning process is to commence as early as possible, either during early adolescence, ideally between the ages of 11 to 14 years, or promptly following the diagnosis of an adolescence-onset disease. It is perceived as per conceptualized that adolescents between the ages of 11 to 12 are developmentally capable of acquiring essential self-care skills and gradually establishing support for transition (14). Secondly, communication among the triad of relationships comprising patients, caregivers, and healthcare providers plays a vital role in devising optimal personalized pathways to facilitate the transitioning process (35). Ideally, there should be a minimum of two direct discussions between these key members of the transition process. The planned process and progress such as readiness for transfer, self-management skills and medical summary should be documented in the medical records for easy referencing by the adult and pediatric team. Thirdly, it has been reported that the successful engagement of adolescents necessitates a team-based approach, with predictors indicating its crucial delivery of high-quality transitional care (13). A clear description of the multidisciplinary team (MDT) ought to be accessible, with the aim of highlighting and tackling the distinct yet equally significant functions among the various MDT members. This is to guarantee the achievement of continuity of care, regardless of the department and services involved. Consequently, healthcare teams participating in the transitioning process must possess suitable training in general adolescent health and childhood onset diseases (16). The key training components to be covered can include: (i) presentation in childhood, knowledge, and approaches to management; (ii) adolescence health and impact on the disease; (iii) skills and knowledge to address the emotional, mental and social issues; (iv) promotion of healthy lifestyle and generic health issues; (v) promotion of self-management and shared decision making; (vi) communication skills with adolescents and caregivers. Thus, funding for training of MDT must be well-planned and allocated. On top of that, policy makers and advocates must guarantee secure funding for dedicated resources to provide uninterrupted clinical care and transition services for adolescents transitioning to adult care. Ideally, the timing of transfer should not be contingent on the availability of resources or the patient’s age, as this could compromise the quality of care for adolescents. Thus, we aspire that the recommendations outlined above for rheumatology-related diseases will serve as a model for the future development of transition care guidelines for PID.

Our study has identified several limitations. Firstly, the opinions of our participating experts cannot be generalized to the entire population of healthcare professionals in the country. It is possible that the experts who responded to the survey were more likely to be actively involved in transition practice, whereas those who did not respond may not be providing adequate support to transition their patients to adult care. Secondly, it is possible that the opinions provided by our participants may be biased toward specific geographical regions. For instance, patients with PID residing in rural areas may encounter unique challenges and have difficulty accessing transition care services. Thirdly, our survey-based data from all experts are subjective in nature and so this is by definition a source of bias. Furthermore, the differing cultural and demographic backgrounds across SEA countries present challenges in directly comparing the work being done in these regions.

## 5 Conclusion

This marks the first multinational survey conducted among SEA PID pediatric experts on the topic of transition practice for patients with PID. The scarcity of structured implementation of transition programs has been shown to contribute to poor quality care for patients with PID as they approach adulthood. Inadequate availability of adult immunologists and specialized centers that can accommodate pediatric conditions were identified as significant barriers to transition for healthcare providers. Conversely, patient/caregiver-related barriers to transition were primarily related to psychosocial factors. Addressing this issue would require additional efforts toward the development of robust best practice guidelines for transition among PID populations and identify outcome measures that can effectively assess the long-term health impact of transition guidance on PID adults in the future.

## Data availability statement

The original contributions presented in the study are included in the article/supplementary material. Further inquiries can be directed to the corresponding author.

## Ethics statement

This study was approved and supported by the Secretariat of Research and Innovation Universiti Kebangsaan Malaysia (UKM) (Project code: UKM PPI/111/8/JEP-2023-120). The study was conducted in full compliance with ethical principles outlined in the Declaration of Helsinki and Malaysian Good Clinical Practice Guideline.

## Author contributions

AA initiated the conceptual framework, provided expert advice for the study design, and supervised the study. CC was responsible for literature review, study design development, data collection and analysis, and drafted the manuscript. AA, AL, LN, II, IH, WL, YZ, NS, RN, FS-O, MC, LN-N-Q, AN, HT, NT, DM, RA, SC and LT reviewed and edited the manuscript. All authors contributed to the article and approved the submitted version.
